# Integration of Signaling Pathways with the Epigenetic Machinery in the Maintenance of Stem Cells

**DOI:** 10.1155/2016/8652748

**Published:** 2015-12-20

**Authors:** Luca Fagnocchi, Stefania Mazzoleni, Alessio Zippo

**Affiliations:** Fondazione Istituto Nazionale di Genetica Molecolare “Romeo ed Enrica Invernizzi”, 20122 Milano, Italy

## Abstract

Stem cells balance their self-renewal and differentiation potential by integrating environmental signals with the transcriptional regulatory network. The maintenance of cell identity and/or cell lineage commitment relies on the interplay of multiple factors including signaling pathways, transcription factors, and the epigenetic machinery. These regulatory modules are strongly interconnected and they influence the pattern of gene expression of stem cells, thus guiding their cellular fate. Embryonic stem cells (ESCs) represent an invaluable tool to study this interplay, being able to indefinitely self-renew and to differentiate towards all three embryonic germ layers in response to developmental cues. In this review, we highlight those mechanisms of signaling to chromatin, which regulate chromatin modifying enzymes, histone modifications, and nucleosome occupancy. In addition, we report the molecular mechanisms through which signaling pathways affect both the epigenetic and the transcriptional state of ESCs, thereby influencing their cell identity. We propose that the dynamic nature of oscillating signaling and the different regulatory network topologies through which those signals are encoded determine specific gene expression programs, leading to the fluctuation of ESCs among multiple pluripotent states or to the establishment of the necessary conditions to exit pluripotency.

## 1. Introduction

Stem cells balance their self-renewal and differentiation potential by integrating environmental signals with the transcriptional regulatory network (TRN) [1–4]. Adult stem cells are generally long-lived quiescent cells, which, upon prodifferentiation stimuli, would give rise to progenitors that will further differentiate into postmitotic mature cells. Controlling the equilibrium between stem cell self-renewal and cell fate specification is indispensable for maintaining tissue homeostasis and the deregulation of these processes would lead to loss of cell identity and tumor initiation [5–7]. In the early embryo, the inner cell mass (ICM) cells are pluripotent and progressively restrict their developmental potential in response to local cues, which direct the formation of the three germinal layers. Defining the molecular mechanisms that govern the establishment of a defined epigenetic program in response to transient signals is fundamental to understand the basis of stem cell specification and reprogramming. The feasibility of isolating and propagating in culture both embryonic and adult stem cells, which can self-renew or differentiate in response to specific signals, allows delineating how extrinsic signals are integrated with the TRN [5, 8–10]. Signaling pathways crosstalk fine-tunes the correct pattern and timing of gene expression by modulating downstream effectors such as transcription factors (TFs), cofactors, and histones modifiers. These modulations are achieved through different mechanisms including differential DNA binding affinities, protein shuttling, posttranslational modifications, and protein-protein interactions. Importantly, the combinatorial DNA binding action of cell type-specific TFs and signal effectors on* cis*-regulatory elements is strongly influenced by the chromatin landscape of a given cell, thus resulting in the establishment of multiple transcriptional programs. In this regard, the dynamic interplay between signaling pathways, TFs, and epigenetic machinery plays a major role in integrating multiple inputs and switching a transient signaling event into a long-lasting phenotypic change.

In this review, we will discuss regulatory mechanisms through which signaling cascade can directly regulate histone modifications, nucleosome occupancy, and chromatin modifying enzymes. We will highlight how these chromatin modifications triggered by extrinsic signaling may affect the TFs binding, the epigenetic state, and the consequent gene expression program of stem cells. Finally, we would underline the critical role of these regulatory circuits to control the cell identity and how their misregulation may initiate pathological events such as tumorigenesis.

## 2. Mechanisms of Signaling to Chromatin 

### 2.1. Signaling Mechanisms Regulating Histone Modifications

The coordinated activation of signaling pathways impacts the epigenetic landscape by targeting TFs, chromatin regulators, or nucleosome occupancy or by directly modifying nucleosomes (Table 1).

Histones are subject to a large set of posttranslational modifications (PTMs) including phosphorylation, acetylation, methylation, ubiquitination, sumoylation, and citrullination, which influence the chromatin structure [11, 12]. The possible combinations of histone modifications differently affect the chromatin accessibility to TFs and determine molecular platforms for recruiting regulatory complexes, which would further modify the chromatin state. Importantly, histone modifications are reversible as opposing modifying enzymes, writers and eraser, introduce or remove the same modifications in response to specific signals [13–16]. Among them, kinases are activated mainly by upstream signaling cascade and they transiently phosphorylate both histone and nonhistone nuclear proteins [17, 18]. The temporal pattern of a certain pathway combined with the cell type-specific chromatin state strongly affects the resulting transcriptional outcome [19]. A large body of data shows that histone phosphorylation influences the deposition of other histone modifications facilitating the recruitment of Histone Acetyltransferases (HATs) while opposing the maintenance of repressive marks. Phosphorylation of histone H3 on serine 10 (H3S10ph) is accomplished in response to different signaling cascades, which activate the downstream kinases such as Rsk2, MSK1/2, IKK*α*, Aurora B, and PIM1 [14, 20–23]. Although the stimulus-induced H3S10ph is transient, it could cooperate with histone acetylation in blocking the binding of the chromodomain containing protein HP1*γ*, thus promoting chromatin remodeling and transcription activation [23]. At enhancers, H3S10 phosphorylation drives the recruitment of MOF, which, by acetylating histone H4, establishes a nucleosome binding platform for the BRD4/P-TEFb complex, thereby stimulating transcription elongation [24]. Other histones phosphorylation is involved in controlling transcriptional switch by mediating histone crosstalk. For example, during androgen receptor- (AR-) dependent gene activation, PKC*β*-mediated H3T6 phosphorylation switches the LSD1 demethylation activity from H3K4 towards H3K9 methyl group [25]. Similarly, the epidermal growth factor- (EGF-) activated tumor-specific pyruvate kinase M2 (PKM2) phosphorylates histone H3 at T11, which triggers dissociation of HDAC3, thus favoring H3K9ac and transcription activation [26].

Taken together, these results illustrate how a kinase-mediated short-lived signal activates a cascade of events, which determines a long-standing output by inducing chromatin modifications and impacting gene expression.

### 2.2. Signaling Mechanisms Regulating Nucleosome Occupancy

Nucleosome organization and higher order chromatin structures package genomic DNA, limiting its accessibility to most of the nuclear factors. Chromatin remodelers are multisubunit complexes that utilize ATP hydrolysis to mobilize nucleosomes and their positioning on the eukaryotic DNA, thereby being essential for modulating chromatin accessibility to transcription factors and RNA Polymerases [27, 28]. In addition, histone chaperones and DNA helicases facilitate histone exchange and the insertion of histone variants into nucleosomes surrounding* cis*-regulatory elements such as promoters and enhancers [29–32]. Many sophisticated mechanisms involve the crosstalk between signaling pathways and chromatin remodelers in order to alter nucleosome occupancy, as a requisite for gene regulation. The steroid hormone receptors interact and recruit SWI/SNF complexes to render the chromatin more accessible. In breast cancer cell, progesterone-activated ERK1/2 phosphorylates both the progesterone-receptor (PR) and the downstream kinase MSK1, forming an active ternary complex, which mediates the phosphorylation of histone H3 at serine 10. This initial step triggers the recruitment of histone modifiers and chromatin remodeling complexes, which ultimately leads to local displacement of histones H1 and H2A/H2B. In this setting, chromatin remodeling is responsible for transcriptional activation of progesterone responsive genes [33–35]. Another study linked nucleosome occupancy at enhancers to androgen receptor (AR) signaling [36]. Apart from nuclear receptors, other signaling pathways have been recently involved in modulating nucleosome occupancy. Specifically, it has been shown that the downstream effectors of the Hippo pathway YAP/TAZ promote transcriptional repression of numerous target genes by stimulating chromatin remodeling. YAP/TAZ interact with the TEAD transcription factor and recruit the NuRD complex on target genes, causing histones deacetylation and increased H3 histone occupancy, thus leading to chromatin compaction [37].

These data illustrate how chromatin remodelers are influenced by environmental signals, which in turn modulate nucleosome occupancy, thereby affecting transcription regulation.

### 2.3. Signaling Mechanisms Regulating Chromatin Modifiers

Signaling pathways can also impact the chromatin state by targeting chromatin modifying proteins. The activation of the Jak2/STAT5 pathway leads to Jak-dependent phosphorylation of STAT5, which causes its dimerization, nuclear translocation, and binding to* cis*-regulatory elements. In addition, Jak2 functions as histone tyrosine kinase by phosphorylating H3Y41 and perturbing HP1*α* binding [38].

Another example of linking signaling pathways with chromatin modifications is represented by the Polycomb and Trithorax group of proteins which act antagonistically in maintaining a specific gene expression state [39, 40]. The H3K27 methyltransferase enzyme EZH2 is the catalytic subunit of the polycomb repressive complex 2 (PRC2) and is targeted by different signals, which can promote or inhibit its enzymatic activity, respectively [41, 42]. The stress-activated p38*α* kinase phosphorylates EZH2 on Thr372 in muscle satellite cells and promotes PRC2-mediated repression of Pax7 during myogenesis. Instead, the prosurvival PI3K-AKT signaling pathway targets EZH2 by inducing Ser21 phosphorylation, which causes the reduction of PRC2 affinity for histone H3. At the same time, AKT-mediated phosphorylation of P300 increases its H3K27-specific acetyltransferase activity, thus participating in switching from a methyl (repressive) towards an acetylated (active) K27 state.

On the other hand, Myeloid/Lymphoid or Mixed-Lineage Leukemia (MLL) group of proteins mediates the trimethylation of histone 3 at lysine 4 (H3K4me3) and are core components of the Trithorax complexes. Multiple MLLs are targeted in response to signaling leading to their PTMs. For example, during the commitment of myoblasts into multinucleated myotubes, p38 MAPK signaling pathway leads to phosphorylation of Mef2d and its interaction with MLL2 complex. This signaling cascade promotes MLL2 targeting to muscle-specific genes leading to their H3K4 trimethylation and transcriptional activation [43].

Overall, the reported examples clearly show that signaling cascades not only influence the activity of transcription factors but also perturb the chromatin state by driving dynamic chromatin changes that impact on the transcriptional program.

## 3. Outcomes of Integrated Signals on Stem Cells Transcriptional and Epigenetic State

**Figure 1:**
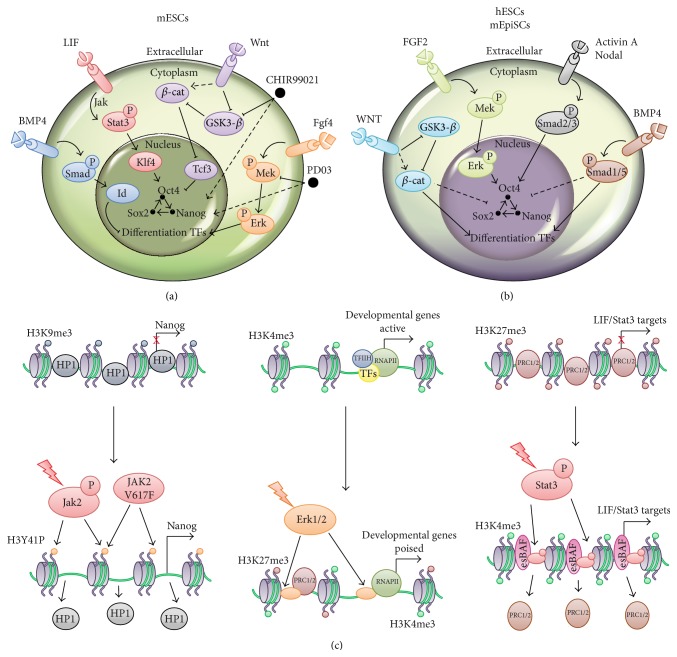
Signaling affecting stem cells identity and their interplay with chromatin. Key signaling pathways and relative factors contributing to the maintenance of mESCs (a) or hESCs/EpiSCs (b) identity or to their differentiation (see details in the main text). Black circles in (a) indicate the two chemicals used in the 2i culturing medium (CHIR99021 and PD03). Solid black arrows and lines indicate positive or negative modulation, respectively. Dashed black lines indicate indirect effects. Colored circles with “P” indicate phosphorylation. (c) Key examples of signaling to chromatin in ESCs. The upper panels are relative to a more differentiated state in which the LIF/Stat3 and Nanog targets are repressed while developmental genes are active. Lower panels, instead, describe embryonic stem cells chromatin features. On the right, effect of Jak2, or its constitutive active form Jak2V617F, on H3Y41P and HP1 loading on chromatin. In the middle, interconnection between Erk1/2 and the loading of PRC2 and RNA polymerase II activity at developmental genes. On the left, interplay between the esBAF complex and Stat3 in regulating LIF/Stat3 signaling pathway targets. Details of each example are reported in the main text.

Beside the examples described so far, developmental signaling pathways are also interconnected with the TRN and influence the chromatin state of stem cells (Figure 1). The developmental signaling, which includes the Wnt/*β*-catenin, Notch, Nodal/Activin, Hippo pathways, and the circadian clock, is involved both in the maintenance of stem cell homeostasis and in inducing cell lineage commitment. In general, their activation triggers the stabilization and the nuclear accumulation of their downstream effectors, which finally influence the expression of their target genes. The downstream effectors, which are activated in a controlled spatiotemporal manner by the external stimuli, provide the competence for a stem cell to adopt a particular cell fate by cooperating with the cell type-specific TFs. This concept is particularly relevant in pluripotent embryonic stem cells (ESCs), in which the same signaling pathways play a key role in the maintenance of self-renewal capacity but are also involved in lineage differentiation. This divergent stem cell responsiveness depends on the fact that signaling pathways target both TRNs and chromatin landscapes. Besides that, the integration of multiple extrinsic signals determines different transcriptional program, thus influencing the cellular response.

### 3.1. Signaling to the Transcriptional Regulatory Network of Embryonic Stem Cells

Both mouse and human ESCs (mESCs and hESCs) are isolated from the transient pluripotent cells of the inner cell mass (ICM) [44, 45]. The two major features that define ESCs consist in their ability to self-renew as well as to differentiate into all the cell lineages in response to developmental cues. This balance is regulated by a specific transcription program, which is centered on the cooperative action of the pluripotency transcription factors Oct4, Sox2, and Nanog (OSN) [46]. OSN targets have been mapped and showed an extensive cobinding in both mESCs and hESCs, suggesting the existence of a common core transcriptional regulatory network (TRN) [47, 48]. Oct4 is a member of the POU family of homeodomain proteins and it is essential for the establishment and maintenance of pluripotency both* in vivo* and* in vitro*. Perturbation of Oct4 transcript level abrogates formation of the ICM [49] and promotes ESCs differentiation [50]. Oct4 heterodimerizes with the high-mobility group box (HMG) family member Sox2 and they cobind distal regulatory elements, thus activating the expression of many pluripotency factors and repressing lineage-specific genes. The synergic action of Oct4/Sox2 in the regulation of key pluripotency factors is underlined by the similar phenotype observed both during blastocyst formation and in cultured ESCs upon the knock-out of the respective genes [51, 52]. Although Nanog is not essential for deriving and maintaining ESCs, it is required for the formation of the ICM. Functionally, Nanog cooccupies most sites with Oct4/Sox2, thus playing a key role in controlling pluripotency in ESCs [53–55]. These core transcription factors control the ESCs transcriptional program by establishing an interconnected regulatory loop in which they influence the gene expression level of each other. This self-sustained transcription regulatory network generates a bimodal transcriptional state of ESCs, which is characterized by the coexistence of transient and exchangeable cellular states. Appropriate levels of the core transcription factors ensure a residence state in which ESCs self-renew. On the contrary, transient perturbation of the positive feedback transcriptional program produces a window of opportunity to exit pluripotency and to initiate cell lineage commitment [55–57]. The ability of OSN to maintain mESCs state is influenced by additional transcription factors such as Klf4, Klf2, Dax1, Nac1, Zfp281, Essrb, Sall4, Tbx3, and Prdm14, which cobind enhancers occupied by OSN [3, 58–60]. Importantly, the OSN-centered regulatory network includes also Stat3, Smad1, and Tcf3, which are the downstream effectors of the LIF, BMP4, and Wnt signaling pathways [3, 61, 62]. These observations underline how the extracellular signals converge on the core TRN, thus participating in the modulation of the stem cell transcriptional program (Figures 1(a) and 1(b)). While LIF leads to phosphorylation of Stat3, which is required to promote self-renewal, BMP4 suppresses differentiation through Smad1-mediated activation of Id genes. Wnt signaling counteracts the transcriptional repressive activity of Tcf3 on pluripotency genes by stabilizing *β*-catenin [63, 64] (Figure 1(a)). However, both hESCs and mouse postimplantation epiblasts derived stem cells (EpiSCs), collectively referred to as “primed” pluripotent stem cells, depend on different signaling pathways for self-renewal, such as FGF/ERK and Activin A/Smad [65, 66] (Figure 1(b)). In particular, both the Wnt/*β*-catenin pathway and the BMP/Smad signaling cascades, which are required to promote mESCs pluripotency, once activated in primed stem cells trigger mesoendoderm lineage commitment. In hESCs, nuclear *β*-catenin cooperates with SMAD2/3, leading to the activation of differentiation genes, thus inducing exit from pluripotency [67]. On the other hand, it has been shown that BMP4/TGF-*β* stimulation induces hESCs and EpiSC to differentiate towards mesoderm [68].

### 3.2. Signaling to Chromatin in Embryonic Stem Cells

Signaling-mediated gene regulation in ESCs could be directly achieved through the modulation of chromatin players and the epigenetic machinery (Figure 1(c)).

In mESCs, a LIF-independent role for Jak signaling has been demonstrated and consists in the phosphorylation of histone H3 on tyrosine 41 (H3Y41). This event leads to a reduction in the binding of heterochromatin protein 1*α* (HP1*α*) on pluripotency genes [69]. In hematopoietic stem cells, mutations leading to the activation of Jak2 correlate with myeloproliferative neoplastic and leukemic transformation. One such mutation is represented by the Jak2V617F allele, which turns on the Jak/STAT pathway without the requirement of activating cytokines [70, 71]. Interestingly, the expression of Jak2V617F in mESCs leads to their cytokine independent self-renewal and is associated with the direct Jak2 signaling to the chromatin. Chemical inhibition of the Jak/STAT pathway in Jak2V617F mESCs leads to the decrease of H3Y41ph levels coupled with increased association of HP1*α* to Nanog promoter, thereby inducing its transcriptional repression. These findings underline the critical role of the direct Jak2 signaling to the chromatin in sustaining self-renewal of both embryonic and hematopoietic stem cells and how its deregulation may cause tumorigenesis [69]. In the same oncogenic setting, mutated Jak2 may also phosphorylate and inhibit PRMT5 preventing histone arginine methylation and favoring uncontrolled haematopoietic progenitor cell expansion [72]. Finally, both Jak2V617F and Jak2K539L, other oncogenic forms of Jak2, cooperate with the histone demethylase JMJD2C in lymphomas, by promoting MYC overexpression [73]. Contrary to the role of Jak2 on chromatin, MAP kinases signaling favors mESCs differentiation through the JNK-mediated H3 Ser10 (H3S10) phosphorylation of its target genes [74].

Other mechanisms of signaling to chromatin involve the modulation of the targeting of chromatin complexes. ERK pathway regulates PRC2 deposition at developmental genes, by phosphorylating the RNA polymerase II at serine 5 and establishing poised domains [75]. The chromatin remodeling complex esBAF is, instead, interconnected with the LIF/Stat3 signaling pathway. Brg1, the ATPase subunit of esBAF, favors the correct targeting of Stat3 onto chromatin by stimulating chromatin remodeling at Stat3 target genes, thus supporting mESCs pluripotency [61]. Similar mechanisms are shared between ESCs and cancer cells and are relevant for tumorigenesis. In ESCs, the pluripotency genes Myc and LIN28 counteract the action of Let-7, which inhibits self-renewal genes [76, 77]. Among others, HMGA2 represents a DNA binding and chromatin modifying protein which regulates both differentiation and stem cell self-renewal [78, 79]. Misregulation of the components of this regulatory circuit has been associated with a wide range of malignancies [80, 81]. Interestingly, in breast cancer cells, inhibition of the MAPK signaling by the Raf kinase inhibitory protein (RKIP) is transduced onto the chromatin where the HMGA2 activity is inhibited, leading to inactivation of proinvasive and prometastatic genes [82]. In hESCs, the Activin A/Smad pathway has been demonstrated to be involved in the correct deposition of H3K4me3 on key developmental genes, through its effectors SMAD2/3, which cooperate with NANOG to recruit DPY30, a subunit of the COMPASS methyltransferase complexes, contributing to the capacity of stem cells to differentiate into specific lineages [83].

### 3.3. Gene Expression Heterogeneity of ESCs and Fluctuating Signaling

Single-cell studies on mESCs showed substantial gene expression heterogeneity with subpopulations of ESCs, which express variegated levels of pluripotency-associated factors [55, 56, 84–87]. The discovery of fluctuating expression levels of pluripotency regulators, which supports the existence of interconvertible ESCs states with different potency to self-renew or differentiate, highlights the key role of sustaining a dynamic transcriptional program in pluripotent cells [87]. Fluctuations in gene expression may depend on multiple factors, which include the structure of the cell TRN, sequential and combinatorial epigenetic regulations, and the integration of signaling pathways.

The TRNs are characterized by recurring regulatory circuits, named network motifs, which define a particular pattern of interconnections, leading to a certain transcriptional outcome [88]. Among them, negative feedback loops and type 1 incoherent feedforward loops may generate oscillatory responses of TFs. Specifically, the ESCs regulatory circuit is characterized by dynamic TFs that regulate each other and autoregulate their own expression through both feedforward and negative feedback loops, thus determining fluctuating states of transcript levels within the ESC population [1, 48, 59, 60, 89–92]. At the posttranscriptional level, microRNAs (miRNAs) play a central role in modulating TRN as the core pluripotency factors OSN and Tcf3 directly bind their loci, thus influencing their expression [93]. Mechanistically, the ES cell-specific cell cycle-regulating (ESCC) miRNAs indirectly activate several self-renewal genes including c-Myc and Lin28 which, by inducing degradation of pre-Let-7 transcripts, inhibit Let-7 opposing effects on ESCs self-renewal [76, 77]. These results suggest that let-7 and ESCC miRNAs act in self-reinforcing loops to sustain the ESCs transcriptional network. The finding that many miRNAs target the pluripotency TFs in ESCs suggests that they may be involved in controlling their fluctuating transcript levels.

Recently, it has been shown that impairment of miRNAs production in ESCs (Dgcr8^−/−^ and Dicer^−/−^ ESCs) resulted in a more homogenous expression of pluripotency factors [87]. In terms of transcription heterogeneity, the Dgcr8^−/−^ ESCs manifest features similar to the so-called 2i ESCs, which mirror the “naïve” or “ground state” of preimplantation epiblast cells [2, 94]. The 2i ESCs are grown in a chemically defined medium which comprises the Mek inhibitor PD03 (PD0325901) and the GSK3 inhibitor CHIRON (CHIR99021), which shield ESCs from prodifferentiation autocrine signaling and reinforce for pro-self-renewing pathways [2] (Figure 1(a)). The Fgf4/Erk cascade drives the transition from naïve pluripotency to a primed state, which is responsive to lineage-specific differentiation signals [95]. GSK3 inhibition reinforces the Wnt pathway by stabilizing *β*-catenin [64, 96–98]. In 2i ESCs, the fluctuating expression of the pluripotency-associated transcription factors is strongly reduced, thus highlighting the crucial role of signaling pathways in modulating transcriptional pulsing in ESCs.

These observations could be explained by considering the intrinsic feature of signaling pathways, which is the* dynamics.* This represents an additional mode of transmitting information, meaning that signaling pathways encode information in the frequency, amplitude, and duration of the signals into the cells [99]. Importantly, cells are able to decode the signaling dynamics by executing different biological responses. Fox example, studying the ERK pathway revealed that different upstream signals trigger divergent dynamic patterns of the same signaling cascade leading to two different cellular fates [100]. In this case, the EGF treatment of PC-12 neural precursors drives a transient ERK activation, which induces cell proliferation, whereas the NGF stimulus triggers a sustained ERK response, culminating in differentiation. The differences in ERK dynamics, in response to those alternative growth factors, depend on the network structure, which encodes the stimulus into a dynamic change of ERK activation. In the EGF pathway, the stimulus activates a SOS-dependent negative feedback loop. Instead, the NGF signal induces a PKC-centered positive feedback loop, thus sustaining ERK activation [101, 102]. The molecular mechanisms through which cells decode the temporal pattern of a certain signaling are poorly understood. Regarding the ERK dynamics, it has been proposed that the interpretation of the temporal signals is depending on network motifs that sense the spatiotemporal changes of the upstream signaling [103–105]. In this case, transient ERK activation leads to the expression of the immediate early gene c-Fos that is rapidly degraded. On the contrary, a persistent nuclear ERK signal drives the accumulation of the effector, which is directly phosphorylated by ERK itself, thus increasing its protein stability.

**Figure 2:**
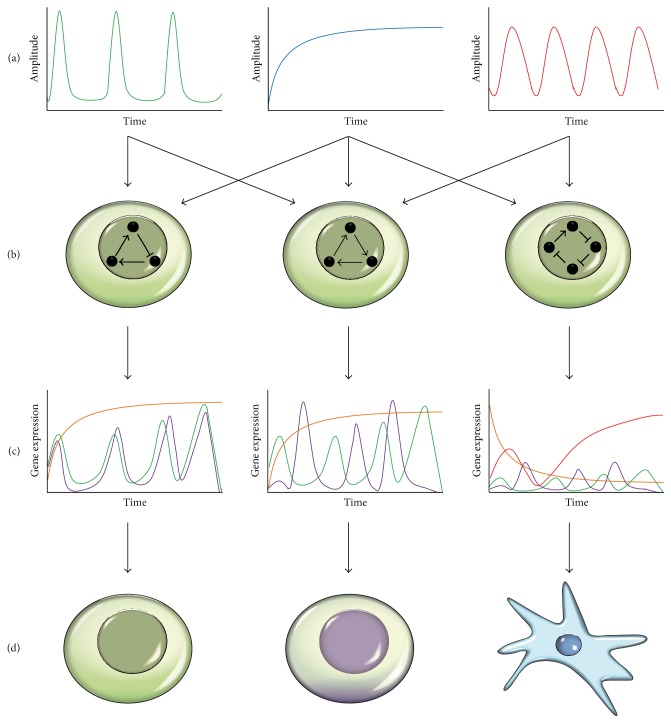
Emergence of gene expression heterogeneity in ESCs and cell fate determination. Gene expression heterogeneity of ESCs is determined by complex multistep mechanisms. (a) Multiple spatiotemporal restricted signals are differentially sensed and integrated by ESCs, leading to signaling pathways activation, which ultimately converges both onto the TRN and directly onto the chromatin. (b) Specific regulatory networks, which involve both TFs and epigenetic regulators, are established in the cell according to their transcriptional and epigenetic landscape and transduce the signals. Arrows and lines indicate positive and negative regulation between factors (black circles), respectively. Negative feedback loops (left) or incoherent feedforward loops (right) may generate oscillatory responses to signals. (c) The result of this integration is the fluctuation of genes expression profiles among cells, which permits ESCs to fluctuate in a continuum of interconvertible pluripotent states and may generate the suitable condition to exit pluripotency and differentiate. (d) The final biological outcome of this process is the establishment of a heterogeneous population of ESCs captured at different pluripotent states (green and purple cells) or the eventual differentiation toward committed cell (blue cell).

In pluripotent stem cells (PSCs), the ERK pathway triggers opposite cellular response: in naïve ESCs, it induces cell lineage commitment while it sustains self-renewal of primed EpiSCs (Figures 1(a) and 1(b)). This striking difference could depend on the dissimilar cellular and epigenetic context of these PSCs but it may also be caused by diverse signaling dynamics, which are decoded differently, thus leading to divergent cellular fates (Figure 2). Few studies addressed this specific point in ESCs but the obtained results clearly showed a link between ERK signaling pathway and fluctuating transcriptional response [2, 94, 106, 107]. The attenuation of the autocrine Fgf4/MAPK signaling induced by either the chemical inhibition of Mek or the genetic targeting of the heparin sulfate proteoglycans reduces the transcriptional fluctuations of pluripotent transcription factors. Although the dynamics of autocrine FGF signaling has not been studied in mESCs, computational modeling, based on the well-studied EGF signaling in other systems, postulates that the oscillatory pattern of Nanog could depend on the dynamics of the regulatory system, including individual cell-specific changes in parameters of FGF autocrine feedback loop and crosstalk with other signaling pathways. The interconnection between MAPK, PI3K/AKT, BMP4/Nodal, and Wnt signaling plays a major role in the maintenance of hESCs [67]. Of note, the dynamics of these signaling pathways have been described to play a major role in controlling ESC pluripotency and reprogramming [108–112]. Among them, the Wnt/*β*-catenin pathway is particularly interesting as its periodic activation favors cell fusion-mediated reprogramming while its sustained stimulation inhibits it [112]. Mechanistically, the activation of the Wnt signaling in the early stage of reprogramming causes a TCF1-dependent inhibitory effect, while its stimulation in the late phase reinforces reprogramming towards PSCs [110, 113]. It would be interesting to evaluate whether a similar fluctuating Wnt signaling pattern may support the maintenance of the naïve state in ESCs. However, recent data showed that *β*-catenin fluctuates in both “primed” (serum + LIF maintained) and naïve (2i + LIF maintained) mESCs [111]. These results are of particular interest considering that in the 2i condition the inhibition of GSK3-*β* should stabilize the endogenous *β*-catenin, thus suggesting that other regulatory circuits may modulate the dynamics of Wnt/*β*-catenin pathway.

More broadly, the observed transcriptional dynamics of pluripotency-associated TFs may reflect the integration of input at the chromatin level including histone modification, chromatin accessibility, the topology of the transcriptional regulatory networks, and activity of autocrine signaling pathways. Despite their importance, the effects of these fluctuating signaling pathways at the transcriptional and chromatin level have not been investigated so far. For example, there are no data regarding the dynamic response of the downstream effectors of the fluctuating signaling pathways in stem cells, nor on the impact on histone modifications at the target genes. Although understanding how cells decode the different dynamical patterns at the molecular level is currently a challenging goal, it is mandatory to better define these regulatory mechanisms in order to clarify their contribution to the maintenance of stem cell identity and pluripotency.

### 3.4. Transcriptional Dynamics in Neural Progenitor Cell Fate Choice

The importance of the integration between signaling dynamics, TRN, and the epigenetic state is well exemplified during cell lineage choice of pluripotent Neural Progenitor Cells (NPCs) in developing nervous system. In the developing telencephalon, the neuroepithelial cells, which represent the earliest NPCs, proceed towards the formation of Radial Glial (RG) cells by the oscillating Notch signaling [114–116]. Asymmetric cell division of polarized RG cells gives rise to immature neurons which would further differentiate into mature neurons and to intermediate progenitors, which go towards cell division in the subventricular zone (SVZ) before fully differentiating. During neurogenesis, it is essential to maintain a certain balance between self-renewing NPCs, proliferating intermediate progenitors, and their commitment towards postmitotic differentiated cells [114]. This goal is achieved, at least in part, by integrating the fluctuating Notch signaling and the transcriptional regulatory circuit of NPCs. In particular, the Notch pathway induces the expression of the bHLH transcription factors Hes1 and Hes5, which are required for the specification of RG cells [117, 118]. Of interest, in neural progenitors, these factors are expressed in an oscillatory manner in response to the fluctuating expression of the Notch ligand Dll1, as well as a consequence of their negative feedback loop. The Hes transcription factors maintain the precursors' multipotency by inhibiting the proneural bHLH factors Ascl1 and Ngn2 [118]. The two bHLH transcription factors Olig1/2 are required to specify the formation of oligodendrocyte progenitor cells and their subsequent differentiation and maturation. Astrocytes fate determination is the result of the interplay between transcription factors, epigenetic modifiers, and environmental signals. Specifically, during neurogenesis, NSCs become responsive to Jak/STAT and BMP signaling pathways, which support astrocyte differentiation, as a consequence of transcription factors-dependent DNA and histone demethylation of the astrocyte-specific genes [119].

The lineage-commitment factors Ascl1, Hes1, and Olig2 play opposite function in sustaining proliferation and cell differentiation of NPCs [120–123]. This contradictory function can be explained by considering their dynamic pattern rather than their relative transcriptional level. Live cell imaging studies have shown that the Notch-dependent fluctuating pattern of Hes1 causes oscillation of Ascl1 and Ngn2 in neural precursors [124–126]. Of importance, by adapting an optogenetic approach to mimic the spatiotemporal pattern of Ascl1 expression in NPCs, it has been demonstrated that periodic oscillations of this TF induce cell proliferation, while its prolonged transcriptional activation triggers lineage commitment towards the formation of neurons [126]. The molecular mechanism through which NPCs differentially interpret the dynamics of Ascl1 gene expression is currently undefined. In addition, it has not been determined which are the different targets that are responsive to this encoded information. Moreover, it has not been investigated so far whether this expression dynamics may be integrated into the chromatin, giving rise to different pattern of histone modifications in the two opposite settings (fluctuating versus sustained transcription).

## 4. Conclusions and Future Perspectives

Over the recent past years, the massive utilization of systems biology techniques and functional genomics increased dramatically our knowledge on the regulatory networks, which control both the maintenance of cell identity and the lineage commitment. Nonetheless, a better understanding of how cells integrate multiple environmental signals and transduce them onto chromatin, in order to modulate gene expression, is still needed.

In this review, we provide multiple evidences, demonstrating how different pluripotent stem cells rely on specific extrinsic cues, which converge on transcriptional and epigenetic networks, thereby determining their cell fate (Figures 1 and 2). Pluripotency is not an invariant state, but rather represents a continuum of states between which cells can fluctuate in response to both extrinsic and intrinsic signals. This concept is supported by both the heterogeneous expression profile of pluripotency factors registered between ESCs subpopulations and the fact that we can capture* in vitro* multiple pluripotent states, hanging on different regulatory networks. Oscillating, spatiotemporal restricted signaling pathways represent a first causative layer for this heterogeneity (Figure 2(a)). Although their role has not been fully addressed yet, the reported example of the cell fate choice in NPCs clearly supports their potentiality in determining fluctuation of downstream TFs activity. The same oscillating signals can affect the choice of cell fate in different manners, according to the topology of the TRNs they are encoded by (Figure 2(b)). Importantly, we reviewed here multiple data, showing how these TRNs in ESCs collectively involve TFs and chromatin players. Therefore, both the transcriptional and epigenetic landscapes should always be taken into consideration when studying how a signal is transduced to regulate genes expression. Integration of multiple oscillating signaling onto different network motifs is responsible for alternative patterns of genes expression (Figure 2(c)), which then determine the cell fate (Figure 2(d)). Since subtle fluctuations of pluripotency factors expression may reflect fluctuations of stem cells through different pluripotent states, similar mechanisms may also favor the exit from pluripotency and cell lineage commitment. Accordingly, mESCs grown in 2i are less heterogeneous in terms of pluripotency genes expression, partially because these fluctuations are reduced by the chemical control of the signaling required to maintain the self-renewal.

Major interest arises from the observations that the same mechanisms may take place during the onset of cell transformation. Cancer stem cells share unique biological features with embryonic and adult stem cells, such as the ability to self-renew, to indefinitely proliferate, and to give rise to aberrant multiple cell progenies. Therefore, the signaling pathways that are important to define stem cell identity have also been demonstrated to play an important role in tumor formation and maintenance. In addition, ESCs specific signatures related to epigenetic features and TFs activity have been found to be common to multiple tumors [127–130], suggesting that regulatory networks, similar to the ones discussed above in ESCs, may be aberrantly activated during tumorigenesis. Their deep understanding is fundamental to unravel the multistep processes that lead to tumor formation and held promise for novel therapeutic targets.

## Figures and Tables

**Table 1:** Summary of different mechanisms of signaling to chromatin.

Mechanism of signaling to chromatin	Signaling pathway	Chromatin target	Functional outcome	Reference
Histone posttranslational modifications	Serum stimulated PIM1 kinase cascade	H3S10 phosphorylation	Recruitment of MOF, which acetylates H4, thus in turn recruiting the BRD4/P-TEFb complex and stimulating transcription elongation	[14]
	Epidermal growth factor (EGF) induced Rsk2 kinase signaling	H3S10 phosphorylation	Recruitment of HAT complexes and rapid acetylation of phosphorylated H3S10	[20]
	Mitogen- and stress-induced MSK1/2 cascade	H3S10 and S28 phosphorylation	Reduced efficiency in inducing mitogen- and stress-induced IE genes	[20]
	Cytokine stimulated IKKa kinase cascade	H3S10 phosphorylation	Regulation of NF-*κ*B-dependent gene expression after cytokine exposure	[22]
	Mitotic Aurora B kinase signaling	H3S10 phosphorylation	Displacement of HP1 from mitotic heterochromatin and gene activation	[23]
	Androgen dependent PKC*β* kinase signaling	H3T6 phosphorylation	Androgen-stimulated gene expression activation, through modulation of LSD1 demethylating activity	[25]
	Epidermal growth factor (EGF) activated PKM2 kinase cascade	H3T11 phosphorylation	Dissociation of HDAC3 from CCND1 and MYC promoters, introduction of H3K9ac, and induction of transcription activation	[26]
	Jak2/STAT5 signaling pathway	H3Y41 phosphorylation	Jak2 acts as histone tyrosine kinase, which phosphorylates H3Y41 and excludes HP1a from chromatin	[38]

Modulation of nucleosome occupancy	Progesterone-activated ERK1/2 signaling	Histones H1 and H2A/H2B	ERK1/2 mediated phosphorylation of the progesterone-receptors, MSK1 and H3S10, which recruit chromatin remodeling complexes leading to the displacement of H1 and H2A/H2B and transcriptional activation of progesterone responsive genes	[33–35]
	Androgen signaling pathway	Nucleosomes	Induction of a nucleosome-depleted state at androgen receptor enhancers, leading to recruitment of histone modifiers, chromatin remodelers, and ultimately gene activation	[66]
	Hippo signaling pathway	Histones H3	The YAP/TAZ/TEAD ternary complex recruits NuRD complex on target genes, leading to histones deacetylation, increased H3 histone occupancy and reduction of chromatin accessibility	[37]

Regulation of chromatin modifiers	Stress-activated p38*α* kinase cascade	EZH2 Thr372 phosphorylation	PRC2-mediated repression of Pax7 during regeneration	[41]
	PI3K-AKT signaling pathway	EZH2 Ser21 phosphorylation	Suppression of EZH2 methyltransferase activity by reducing its binding to histone H3 and derepression of silenced genes	[42]
	p38 MAPK signaling pathway	MLL complexes	The signaling cascade leads to phosphorylation of Mef2d, which interacts with MLL complex, targeting it to specific genes that are activated during myogenesis	[43]
